# Genomic amplifications identified by circulating tumor DNA analysis guide prognosis in metastatic castration-resistant prostate cancer

**DOI:** 10.3389/fonc.2023.1202277

**Published:** 2024-02-21

**Authors:** Toros A. Dincman, Joseph A. Q. Karam, Antonio Giordano, Hong Li, Leylah M. Drusbosky, Theodore S. Gourdin, Philip H. Howe, Michael B. Lilly

**Affiliations:** ^1^ Department of Medicine, Division of Hematology and Oncology, Medical University of South Carolina, Charleston, SC, United States; ^2^ Hollings Cancer Center, Medical University of South Carolina, Charleston, SC, United States; ^3^ Department of Biochemistry and Molecular Biology, College of Medicine, Medical University of South Carolina, Charleston, SC, United States; ^4^ Medical Oncology, Dana-Farber Cancer Institute, Boston, MA, United States; ^5^ Medical Oncology, Harvard Medical School, Boston, MA, United States; ^6^ Department of Public Health Sciences, University of California- Davis, Davis, CA, United States; ^7^ Guardant Health, Redwood City, CA, United States

**Keywords:** prostate cancer, metastasis, castration-resistant prostate cancer, androgen receptor, PIK3CA, aneuploidy, genomic amplification, circulating tumor DNA

## Abstract

**Purpose:**

Analysis of circulating tumor DNA (ctDNA) in patients with metastatic prostate cancer (mPC) provides an opportunity to identify and monitor genomic alterations during a patient’s treatment course. We evaluated whether the presence of specific gene amplifications (GAs) and plasma copy number (PCN) alterations are associated with disease features.

**Methods:**

This is a single-institution retrospective study of patients with mPC who underwent ctDNA profiling using Guardant360^®^ (Guardant Health Inc.). This test identifies single nucleotide variants (SNVs) and GAs of select genes by next-generation sequencing. A total of 155 men with mPC were studied. Patients were stratified by GA status. The Kaplan-Meier method and multivariate cox regression models were used to estimate overall survival (OS) or failure-free survival (FFS) from either the date of GA detection or the initiation of systemic therapy. The chi-square test was used to evaluate associations between clinical factors and GAs.

**Results:**

The presence of liver and/or lung metastases was associated with GAs of *BRAF, CDK6, PI3KCA*, and *FGFR1*. Survival analyses were completed on a subset of 83 patients with metastatic castration-resistant prostate cancer (mCRPC). Median OS was improved in patients with 1 GA compared to patients with ≥2 GAs, whether determined from the date of initial GA(s) detection (14.9 mo vs. 8.9 mo) or date of therapy initiation nearest to GA detection (16.7 mo vs. 9.0 mo). Patients without GAs had not reached median OS. Patients with androgen receptor (*AR*) GA only were also found to have better median OS compared to patients with *AR* GA plus at least one other additional GA (19.3 mo vs. 8.9 mo). Patients with *PIK3CA* GA had significantly lower median OS compared to patients with GAs that did not have a *PIK3CA* GA (5.9 mo vs. 16.0 mo). In patients with *AR* and/or *MYC* GA(s), median OS improved in those with reduced *AR* or *MYC* PCN during therapy compared to those without such a reduction (25.1 mo vs. 15.9 mo).

**Conclusions:**

The association of select GAs with survival provides an additional tool for assessing mCRPC prognosis and informing management. Serial monitoring of ctDNA GAs is also useful to guide prognosis and therapeutic response.

## Introduction

Prostate cancer (PC) remains the most diagnosed malignancy and the second leading cause of cancer-related death among men in the United States. Between 2014 and 2019, prostate cancer incidence has increased by 3% per year (1, 2). Although most men diagnosed with PC have local disease, recurrences frequently occur (3, 4). Furthermore, diagnosis of metastatic prostate cancer (mPC) has been modestly increasing in the context of changing screening practices and is a dynamic disease with a poor prognosis, particularly as it progresses to castration-resistant prostate cancer (CRPC) (5–8). Prostate cancer has the propensity to develop resistance across various treatment paradigms, which include androgen axis targeting agents and chemotherapy. Thus, uncovering the genomic phenotypes as the disease evolves is critical to understanding patient prognosis, treatment sequencing, and developing novel therapeutics. Circulating tumor DNA (ctDNA) provides an essential tool for understanding such genomic phenotypes, guiding therapeutic decisions, and characterizing tumor heterogeneity in a cost-effective manner. It poses minimal patient risk and little technical challenge compared to (repeated) biopsy of tumor tissue (8–10).

Recently, the use of ctDNA analysis has increased, enhancing our understanding of PC progression and helping to guide clinical management (9). An analysis of more than 500 patients with metastatic CRPC (mCRPC) identified frequent androgen receptor (*AR*) and *MYC* alterations associated with clinical outcomes, such as overall survival (OS) and failure-free survival (FFS) (11). Several *AR* mutations that are associated with resistance to anti-androgen axis therapies were identified by ctDNA analysis and can guide therapeutic decision-making (12). Although distinct from ctDNA, circulating cell-free DNA (cfDNA), which consists of ctDNA and hematopoietic cell-derived DNA, is elevated in patients with mPC and can also serve as a marker of therapeutic response. For example, decreased cfDNA levels were associated with improved outcomes in patients harboring mutations in genes involved in homologous recombination and demonstrating clinical response to poly(ADP)ribose polymerase (PARP) inhibitor (13). Similarly, cfDNA levels were reduced in patients receiving taxane therapy, and that reduction was associated with both radiological progression-free survival (rPFS) and OS (14). Finally, ctDNA levels were associated with rPFS in mCRPC patients in the A.MARTIN phase II study of abiraterone with or without pan-AKT inhibitor ipatasertib (15).

In patients with mPC, the genomic landscape remains fluid throughout the treatment course. ctDNA can be used to profile somatic mutations and genomic amplifications (GAs) with their corresponding variant allele frequency (VAF) plasma copy number (PCN) during any stage of the treatment course. GAs may be representative of either chromosomal duplication and/or gene-specific amplification, which is particularly interesting because aneuploidy is a relevant feature of aggressive prostate cancers that may be more likely to be lethal (16). Copy number alterations frequently occur in the form of loss of regions, with the loss of tumor suppressors *CHD1*, *RB1*, *TP53* and *PTEN* as the most common alterations (17). A recently published copy number alteration analysis from 300 patients in the androgen deprivation therapy control arm of the STAMPEDE trial showed that loss of segments of chromosome 5 containing *CHD1* and amplifications in segments of chromosome 8 containing *MYC* were associated with a higher burden of copy number alterations. Copy number alteration burden was associated with a statistically significant increase in metastasis at diagnosis, risk of progression, and death (18). This follows prior demonstration of the presence of *AR,* or *MYC,* or *BRAF* amplifications (as detected by ctDNA) are associated with worse OS (11). Furthermore, we previously demonstrated that elevated carcinoembryonic antigen (CEA) is associated with higher rates of liver metastases and increased copy number alterations of select genes (19).

The prognostic significance of GAs and PCN detected prior to or serially through the course of treatment in a cohort of patients remains to be fully characterized. The majority of ctDNA assays do not commonly report on actionable deletions, germline mutations, or less frequent GAs such as chromosomal rearrangements (20). This limitation led us to focus on whether GAs and PCN measured through ctDNA analysis alone can inform us on clinical outcomes and therefore continue to build from prior studies. To investigate the associations between GAs and PCN with specific clinical factors and treatment outcomes, we conducted a retrospective analysis of patients at our institution with mPC who harbor GAs.

## Methods

### Patient selection

**Figure 1:**
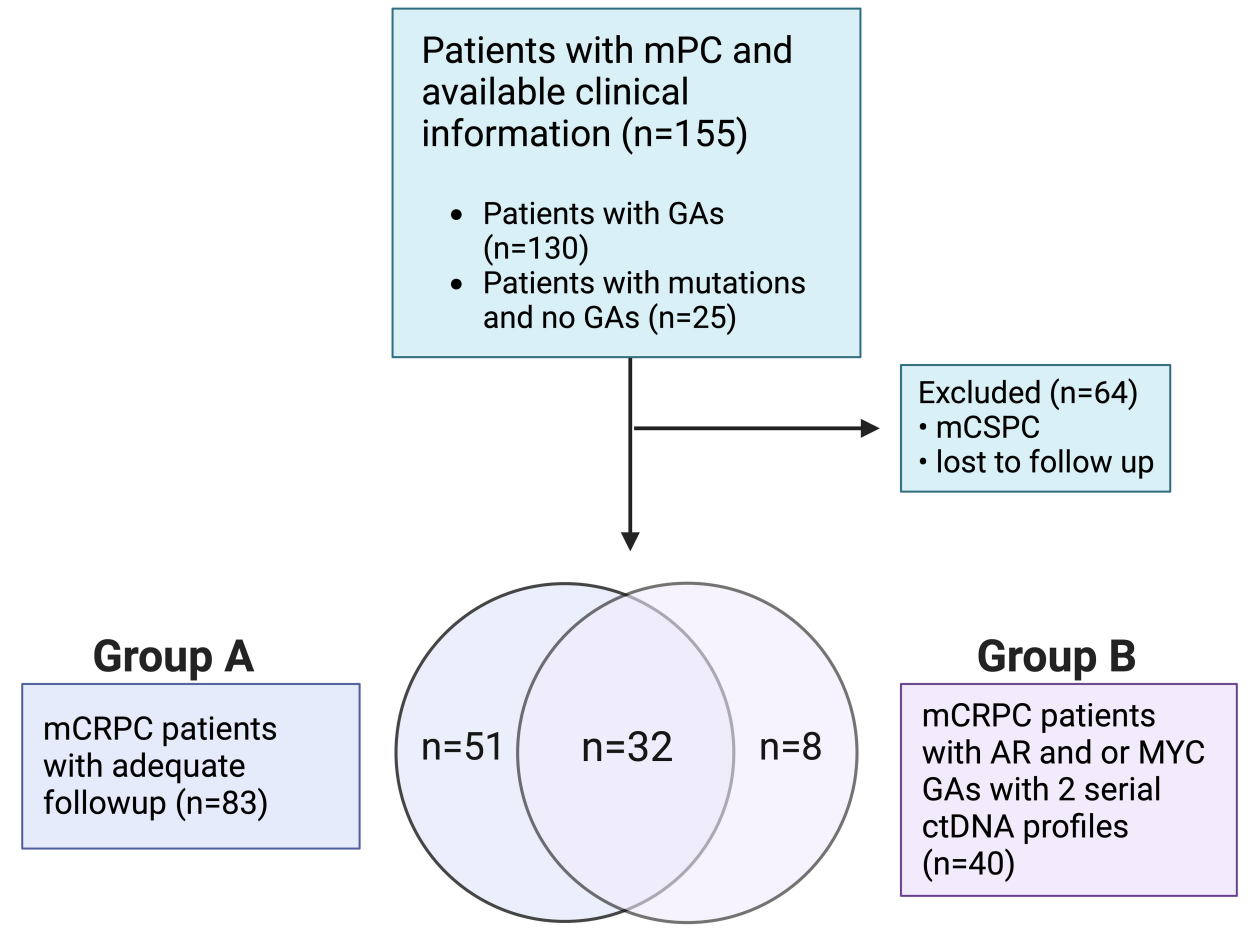
Retrospective study design flowchart.

Patients with mPC who were seen at the Medical University of South Carolina’s Hollings Cancer Center (Charleston, SC) from March 2015 through March 2020 and underwent ctDNA evaluation using the Guardant360® platform (Guardant Health Inc., Redwood City, CA) were eligible for analysis (21). Eligible patients were de-identified, and demographic, clinical, and corresponding ctDNA data were collected. Patients with one or more GAs (i.e., increase in PCN) were selected for downstream analysis. Our cohort (**Figure 1**) included mPC patients with a ctDNA-detectable increase in PCN in at least one gene. Collected clinical data included Gleason score at diagnosis, prostate-specific antigen (PSA), hemoglobin (Hgb), metastatic disease present at diagnosis, sites of metastases, castrate-resistant status, Eastern Cooperative Oncology Group (ECOG) performance status, and systemic therapies received prior to collection of plasma for ctDNA analysis. Metastatic sites included bone only, lymph node only, liver/lung only, bone and lymph node, and liver/lung with other sites. Patients who had been diagnosed with mCRPC were divided into two groups. Patients in Group A (**Figure 1**) had evaluable survival and systemic therapy time points in addition to ctDNA analysis. Overall survival was defined as duration from ctDNA sample collection or treatment start date to study end date or death. Failure-free survival was defined as duration from treatment start date to date treatment was last administered or death. Patients in Group B had detectable *AR* or *MYC* amplification and two serial ctDNA analyses. The conduct of this study was approved by the institutional review board of the Medical University of South Carolina.

### ctDNA profiling

Blood samples for subsequent ctDNA analysis were collected from patients at scheduled clinic visits as part of their routine care. Next-generation sequencing (NGS) of plasma ctDNA (i.e., liquid biopsy) was completed as previously described by Guardant Health (Guardant360®), a College of American Pathologists (CAP)-accredited and Clinical Laboratory Improvement Amendments (CLIA)-certified laboratory (21). Briefly, cfDNA was extracted from whole blood collected in 10-mL Streck® tubes using the QIAmp Circulating Nucleic Acid Kit (Qiagen, Inc). Hybrid-capture sequencing libraries were prepared for up to 30ng cfDNA and labeled with non-random oligonucleotide barcodes (IDT, Inc.), followed by library preparation, hybrid capture enrichment (Agilent Technologies, Inc.), and sequencing at 15,000X read depth of the critical exons in the targeted panel by paired-end synthesis (NextSeq 500 and/or HiSeq 2500, Illumina, Inc.). Bioinformatics analysis and variant detection were performed as previously described (22). NGS data were interpreted by N-of-One, Inc. (Lexington, MA). Over the course of the study, the panel composition expanded from 68 to 70 to 73 to 74 genes. In the 68-gene panel, coverage of gene amplifications included 16 genes. The 74-gene panel includes coverage of 18 gene amplifications. Most samples in this study were tested using the 74-gene panel. All gene amplifications analyzed for this study were sufficiently covered by all iterations of the Guardant ctDNA assay during the study period.

### Statistical analyses

Descriptive statistics were used to summarize all baseline patient characteristics, frequency of GAs, and missense/frameshift mutation frequencies. Chi-square analyses and Odds Ratios were used to evaluate for associations between select clinical factors and GAs. The Kaplan-Meier method was used to estimate OS and FFS outcomes in patients with mCRPC from the time points indicated to either the end of the study (OS), final administration of treatment (FFS), or death (OS, FFS). Survival analyses for patients in Group A were stratified by the presence of one or more than one GA at the time of initial GA detection. Univariable Cox proportional hazards regression was used to identify associations of clinical variables and GAs with OS and FFS. These clinical variables included age, race, metastases at the time of diagnosis, PSA, Hgb, ECOG performance status, metastatic sites, therapy administered prior to ctDNA profiling, and select GAs. Systemic therapies evaluated included abiraterone, enzalutamide, docetaxel, cabazitaxel, carboplatin, sipuleucel-T, and radium-223 in which a p-value less than or equal to 0.2 was used as the initial variable selection criteria. Then multivariable Cox regression models were fit in which a forward variable selection approach was used to generate the final clinical factors which have significant impact on outcomes. Patients in Group B were categorized as either having a “Response” or “No Response”. The term “Response” was defined as having a 10% reduction in *AR* PCN in the ctDNA analysis completed after the initial detection of *AR* amplification. If these variables remained the same or increased, patients were categorized as having “No Response”. Statistical tests were 2-sided with significance defined as p≤ 0.05.

## Results

### Study design and patient characteristics

Among mPC patients seen at our institution, a total of 130 out of 155 tested patients had at least one GA (**Figure 1**). Baseline clinical variables of the study-eligible patient population are detailed in **Table 1** and are reflective of the time of ctDNA analysis and initial GA detection. The median age was 71 years (range 46-91 years), and 36.8% of patients were African American. Almost three-quarters of patients (72.9%) had a Gleason score of 7-10, and the median PSA was 46.2 ng/ml (range 0.1-6000 ng/ml). Nearly half of the patients had metastasis at diagnosis (42.6%). Most patients had mCRPC (87.1%). Select systemic therapies included the androgen axis-targeting drugs abiraterone (47.1%) and enzalutamide (43.9%) and chemotherapy such as docetaxel (44.5%), cabazitaxel (22.6%), and carboplatin (14.2%). Of note, carboplatin was administered in combination with either docetaxel or cabazitaxel and not as a single agent. Sipuleucel-T and Radium-223 were also administered in 15.5% and 10.3% of patients, respectively.

**Table 1:** Baseline characteristics of the mPC cohort.

CHARACTERISTIC	STATISTIC	RESULT
Age, y	Mean (SD)	70.5 (8.3)
	Median (range)	71 (46-91)
Race	No. (%)	
Caucasian	No. (%)	94 (60.6)
African-American	No. (%)	57 (36.8)
Other	No. (%)	4 (2.6)
GS at diagnosis	No. (%)	
5	No. (%)	1 (0.6)
6	No. (%)	10 (6.5)
7	No. (%)	32 (20.6)
8	No. (%)	20 (12.9)
9	No. (%)	46 (29.7)
10	No. (%)	15 (9.7)
Unknown	No. (%)	31 (20.0)
Metastasis at diagnosis	No. (%)	66 (42.6)
CRPC	No. (%)	135 (87.1)
PSA, ng/ml	Mean (SD)	338.3 (949.0)
	Median (range)	46.2 (0.1 -6000)
HGB, g/dl	Mean (SD)	11.3 (2.2)
	Median (range)	11.5 (6.4 - 16.0)
ECOG Performance Status	No. (%)	
0	No. (%)	46 (29.7)
1	No. (%)	79 (51.0)
2	No. (%)	18 (11.6)
3	No. (%)	10 (6.4)
Unknown	No. (%)	2 (1.3)
Metastatic Sites	No. (%)	
Bone	No. (%)	141 (91.0)
LN/Soft Tissue	No. (%)	104 (67.1)
Liver/Lung	No. (%)	34 (21.9)
Bone + LN/Soft Tissue	No. (%)	92 (59.4)
All Sites	No. (%)	21 (13.5)
Prior Systemic Therapy	No. (%)	
Abiraterone	No. (%)	73 (47.1)
Enzalutamide	No. (%)	68 (43.9)
Docetaxel	No. (%)	69 (44.5)
Cabazitaxel	No. (%)	35 (22.6)
Carboplatin	No. (%)	22 (14.2)
Sipuleucel-T	No. (%)	24 (15.5)
Radium-223	No. (%)	16 (10.3)

### Association between certain GAs and visceral metastases

**Figure 2:**
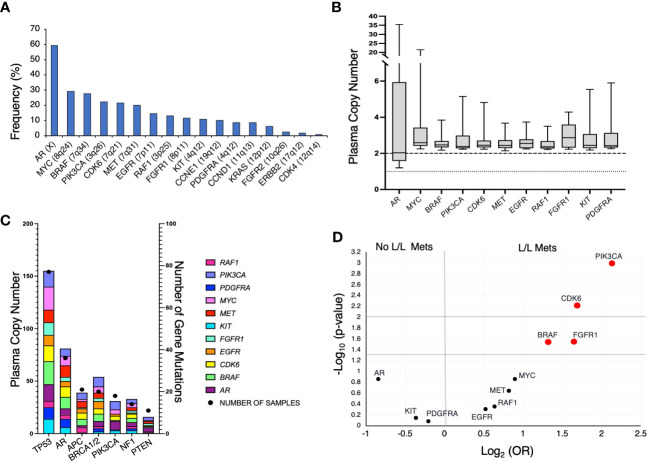
**(A)** Frequency of genomic amplifications (GAs) in our mPC cohort at time of initial identification of increased gene plasma copy number. **(B)** Distribution of PCN in GAs present in at least 10% of patients. Dotted line represent PCN of 1 to illustrate baseline for *AR* and dashed line at PCN of 2 to represent baseline for all described other genes. **(C)** Frequency of mutations and co-occurring GAs. X-axis represents the commonly occurring mutations in cohort A, left Y-axis represents the frequency of GAs, and right Y-axis represents the number of samples that harbored the specified gene mutation listed on X-axis. **(D)** Volcano plot depicting GAs present in at least 10% of patients and association with liver/lung metastases (L/L Mets).

At the time of initial detection of at least one GA, the most frequently occurring GA was in *AR* (**Figure 2A**; 59.2%), followed by *MYC* (29.2%), *BRAF* (27.7%), *CDK6* (22.3%), *PIK3CA* (21.5%), and *MET* (20.0%). Given the relative genomic positions of *BRAF* (7q34), *CDK6* (7q21.2), and *MET* (7q31.2) genes on chromosome 7, these GAs frequently co-occur. For instance, 69% of *CDK6* GAs co-occur with *BRAF* GAs, and 88.5% of *MET* GAs co-occur with *BRAF* and/or *CDK6* GAs. Of the GAs, *AR* had the largest range in plasma copy number (PCN) (1.2 – 35.4), but a median PCN of 2.03 (**Figure 2B**). Of all other identified GAs occurring in at least 10% of patients, the median PCN ranged from 2.37 (*RAF1*) to 2.87 (*FGFR1*) (**Figure 2B**).

We also evaluated the frequency of GAs co-occurring with select mutations. *TP53* was the most frequently mutated gene (**Figure 2C**). Interestingly, despite *AR* amplification being the most frequently occurring GA, patients harboring *TP53* mutations had a greater frequency of concurrent *MYC* and *BRAF* amplifications. Furthermore, patients with mPC with *APC* mutations rarely had the two most frequently occurring GAs, *AR* and *MYC*.

In patients with mPC, liver and/or lung metastases are associated with poorer prognosis and may be indicative of more aggressive mPC subtypes such as neuroendocrine prostate cancer (NEPC) (19, 23). We evaluated whether GAs that occurred in at least 10% of patients were associated with the incidence of liver/lung metastases present at the time of initial GA detection (**Figure 2D**). Liver/lung metastases were found to be significantly associated with amplification of *BRAF* (OR 2.51, 95%CI 1.09 – 5.81), *CDK6* (OR 3.23, 95%CI 1.36 – 7.93), *PIK3CA* (OR 4.37, 95%CI 1.79 – 10.67), and *FGFR1* (OR 3.13, 95%CI 1.1 – 8.95). *CDK6* is frequently co-amplified with *BRAF*, given the relative proximity of its genomic position. Thirty-three percent of patients with *CDK6* GA without co-occurring *BRAF* GA had liver/lung metastases.

### Increased number of GAs associated with patient survival

**Figure 3:**
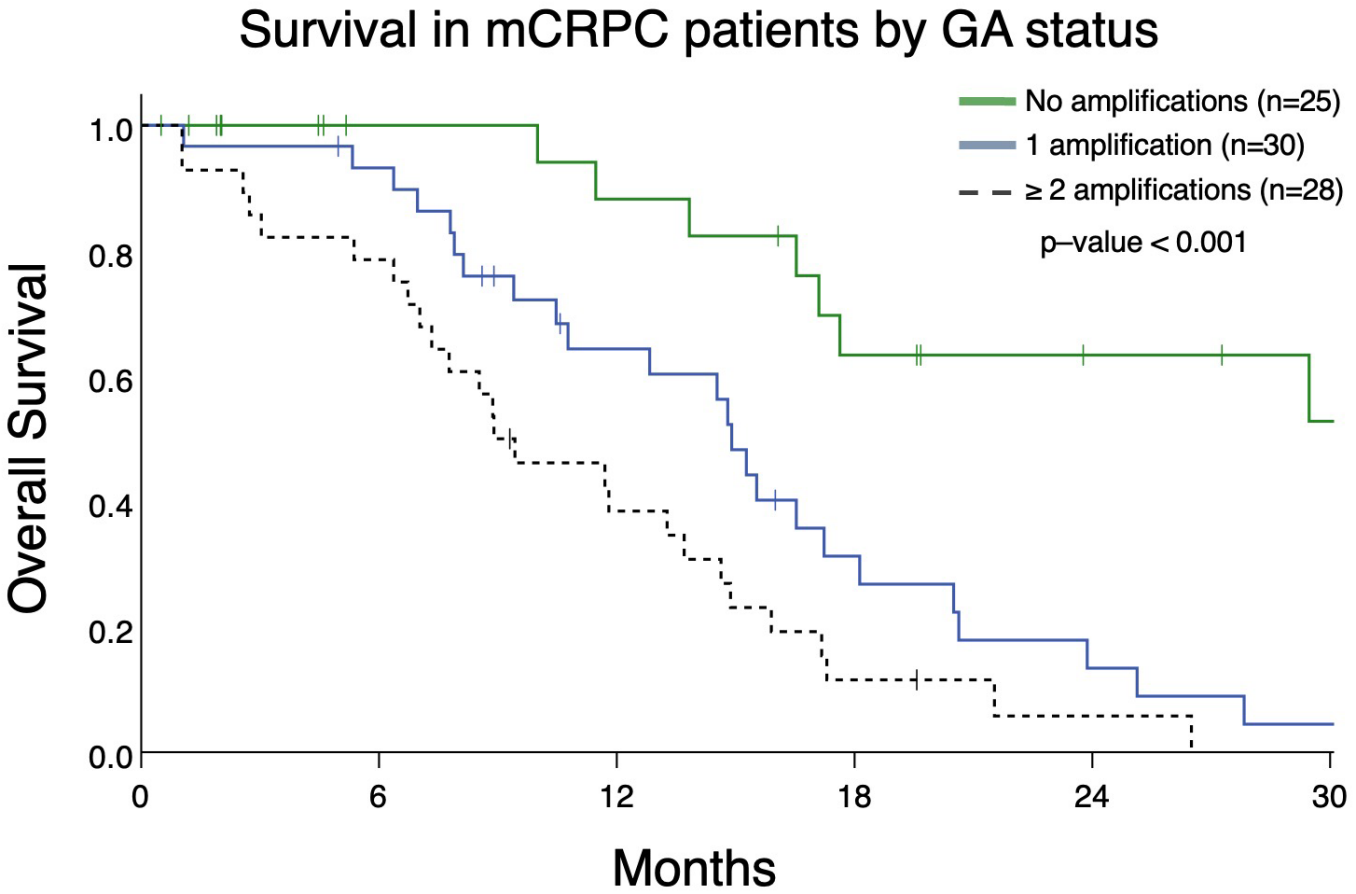
Overall survival in patients with mCRPC (Group A) from the date of initial detection by ctDNA assay in patients with no GAs, only 1 GA, or with ≥ 2 GAs. Patients with no GAs had greater survival (median survival not reached) compared to only 1 GA (median survival 14.9 months) or ≥ 2 GAs (median survival 8.9 months; p<0.001).

**Figure 4:**
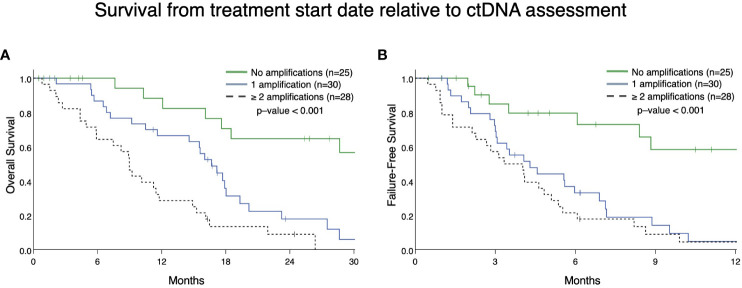
Overall survival **(A)** and failure-free survival **(B)** in Group A from the date of the nearest systemic therapy initialization. **(A)** Patients with mCRPC with ≥ 2 GAs had reduced survival (median survival in patients with one GA was 16.7 months vs. 9.0 months in patients with ≥ 2 GAs; p <0.001). Median survival for no GAs was not reached. **(B)** Patients with mCRPC with 1 or ≥ 2 GAs had reduced failure-free survival (median FFS in patients with one GA was 4.3 months vs. 3.3 months in patients with ≥ 2 GAs; p < 0.001) compared to patients with no GAs (median FFS not reached). There was no statistical difference in FFS between patients with only 1 GA compared to ≥ 2 GAs (p=0.28).

From patients in this cohort, we then identified 83 patients with mCRPC and clinically evaluable complete treatment records (Group A, **Figure 1**). Patients were stratified by whether they had no GAs, one GA, or two or more GAs at the time of ctDNA analysis. There was no statistically significant difference in the mean number of therapies prior to ctDNA analysis amongst these three stratified groups. This analysis demonstrated that patients with two or more identifiable GAs had a poorer OS from the time of initial GA detection than those with only one GA. Median OS was not reached for patients with no GAs, was 16.4 months for patients with only one GA, and 9.4 months for patients with ≥2 GAs (**Figure 3**). Survival analysis was extended to include OS and FFS from the date of systemic treatment initiation nearest to the date of ctDNA analysis. OS and FFS were poorer in patients with ≥2 GAs than in those with only one or no GA. Median OS in patients with one GA was almost double that in patients with ≥2 GAs (16.7 vs. 9.0 months; p-value < 0.001) (**Figure 4A**). Median OS in patients with no GAs was not reached at the time of analysis. Median FFS was significantly different when comparing patients with 1 (4.3 months), or with ≥2 GAs (3.3 months), to patients with no GAs (median OS not reached, p-value < 0.001) (**Figure 4B**).

**Figure 5:**
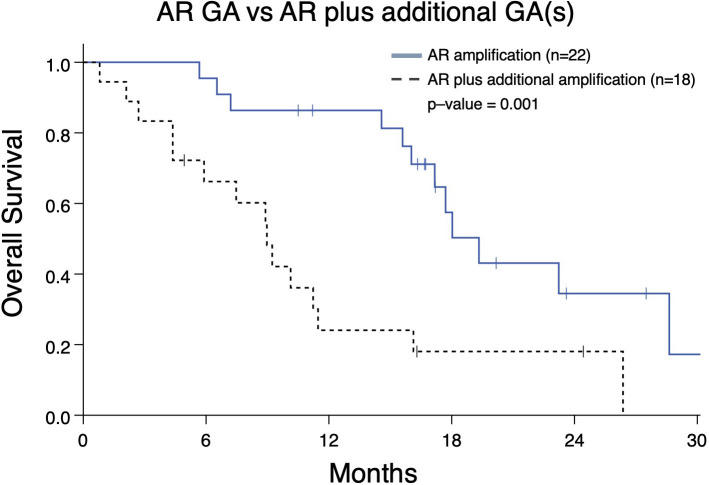
Overall survival in mCRPC patients (Group A) from the date of the nearest systemic therapy initialization with that have either an *AR* genomic amplification (GA) only vs patients with an *AR* GA as well as at least one additional GA in another gene. Patients with *AR* GA only had greater median survival (19.3 months) compared to patients with *AR* GA plus at least one GA (median survival 8.9 months; p=0.001).

As demonstrated above, the majority of detected GAs were in *AR* (**Figure 2A**). Given our prior work demonstrating that presence of an *AR* GA being associated with worse prognosis (11), we next determined whether *AR* plus at least one other GA (designated as “*AR* Plus”) had a difference in OS compared to patients having exclusively an *AR* GA. Patients with only an *AR* GA have a significantly better median OS compared to patients with “*AR* Plus” GAs (19.3 vs 8.9 months; p-value < 0.001) (**Figure 5**).

Cox regression univariate analysis of group A patients indicates that reduced OS may be present with amplifications in *MYC*, *BRAF*, *PIK3CA*, *CDK6*, *MET2*, *EGFR*, and *RAF1* (**Table 2**). However, when adjusted for significant covariates in a multivariate analysis, genomic amplification of *PIK3CA* remains significantly associated with worse survival in the final model (**Table 2**). Consistent with the Kaplan-Meier survival analysis, patients with one GA (HR 3.84 [1.45 – 10.16], p-value <0.01) and ≥2 GAs (HR 7.15 [2.50 – 20.46], p-value <0.001) were also significant in the final multivariate regression model as well. *PIK3CA* is associated with worse FFS in multivariate analysis as well (**Table 3**). Abiraterone prior to ctDNA analysis was shown to be prognostic for FFS (**Table 3**).

**Table 2:** Association of select variables in Group A with overall survival as determined by date of systemic treatment initiation nearest to time of ctDNA analysis.

Variable	Categorization	No.	Univariate Analysis		Multivariate Analysis	
			Hazard Ratio (95% CI)	p-value	Hazard Ratio (95% CI)	p-value
Age, y	Continuous	83	0.99 (0.96 – 1.03)	0.88		
Race	Caucasian or African-American	82	0.98 (0.56 – 1.72)	0.94		
Metastasis at diagnosis	Yes or No	83	1.07 (0.60 – 1.89)	0.83		
PSA, ng/ml	Continuous	82	1.0 (1.0003 – 1.0011)	<0.01		
HGB, g/dl	Continuous	79	0.80 (0.69 – 0.92)	<0.01	0.80 (0.69 – 0.93)	<0.01
ECOG Performance Status	≥1 or 0	81	2.54 (1.19 – 5.45)	<0.05		
Metastasis Site						
Bone	Yes or No	83	2.37 (0.74 – 7.62)	0.15		
LN/Soft Tissue	Yes or No	83	1.71 (0.92 – 3.19)	0.09		
Liver/Lung	Yes or No	83	1.31 (0.64 – 2.70)	0.47		
Bone + LN/Soft Tissue	Yes or No	83	1.81 (1.01 – 3.26)	<0.05		
All Sites	Yes or No	83	1.41 (0.60 – 3.32)	0.43		
Prior Systemic Therapy						
Abiraterone	Yes/No (%Yes)	83 (44.6)	1.87 (1.07 – 3.27)	<0.05		
Enzalutamide	Yes/No (%Yes)	83 (44.6)	1.00 (0.57 – 1.75)	0.99		
Docetaxel	Yes/No (%Yes)	83 (50.6)	1.31 (0.74 – 2.31)	0.35		
Cabazitaxel	Yes/No (%Yes)	83 (21.7)	1.95 (1.08 – 3.55)	<0.05		
Carboplatin	Yes/No (%Yes)	83 (18.1)	1.28 (0.66 – 2.51)	0.47		
Sipuleucel-T	Yes/No (%Yes)	83 (18.1)	0.90 (0.44 – 1.87)	0.79		
Radium-223	Yes/No (%Yes)	83 (13.3)	0.87 (0.34 – 2.22)	0.78		
Gene Amplification						
AR	Yes or No	83	1.43 (0.81 – 2.53)	0.22		
MYC	Yes or No	83	2.58 (1.40 – 4.73)	<0.01		
BRAF	Yes or No	83	2.31 (1.23 – 4.34)	<0.01		
PIK3CA	Yes or No	83	5.98 (2.87 – 12.46)	<0.01	3.05 (1.39 – 6.73)	<0.01
CDK6	Yes or No	83	3.24 (1.60 – 6.59)	<0.01		
MET2	Yes or No	83	2.51 (1.30 – 4.87)	<0.01		
EGFR	Yes or No	83	3.53 (1.56 – 8.02)	<0.01		
RAF1	Yes or No	83	2.28 (0.96 – 5.38)	0.06		
FGFR1	Yes or No	83	2.34 (0.84 – 6.54)	0.11		
KIT	Yes or No	83	2.64 (1.03 – 6.80)	<0.05		

Assessed by univariate and multivariate Cox proportional hazards models. Only variables found to be statistically significant in the final multivariate model have reported hazard ratio and p-value.

**Table 3:** Association of select variables in Group A with Failure-Free Survival as determined by date of systemic treatment initiation nearest to time of ctDNA analysis.

Variable	Categorization	No.	Univariate Analysis		Multivariate Analysis	
			Hazard Ratio (95% CI)	p-value	Hazard Ratio (95% CI)	p-value
Age, y	Continuous	83	1.00 (0.96 – 1.03)	0.73		
Race	Caucasian or African-American	82	1.05 (0.60 – 1.84)	0.87		
Metastasis at diagnosis	Yes or No	83	1.12 (0.63 – 2.00)	0.70		
PSA, ng/ml	Continuous	82	1.00 (1.001 – 1.002)	<0.01	1.001 (1.001 – 1.002)	<0.01
HGB, g/dl	Continuous	79	0.83 (0.72 – 0.97)	<0.05		
ECOG Performance Status	≥1 or 0	81	2.87 (1.34 – 6.15)	<0.01	2.46 (1.14 – 5.33)	<0.05
Metastasis Site						
Bone	Yes or No	83	2.00 (0.62 – 6.42)	0.25		
LN/Soft Tissue	Yes or No	83	1.39 (0.75 – 2.59)	0.30		
Liver/Lung	Yes or No	83	1.41 (0.68 – 2.90)	0.35		
Bone + LN/Soft Tissue	Yes or No	83	1.60 (0.89 – 2.89)	0.12		
All Sites	Yes or No	83	1.48 (0.63 – 3.47)	0.37		
Prior Systemic Therapy						
Abiraterone	Yes/No (%Yes)	83 (44.6)	2.44 (1.37 – 4.34)	<0.01	2.34 (1.26 – 4.37)	<0.01
Enzalutamide	Yes/No (%Yes)	83 (44.6)	0.85 (0.49 – 1.49)	0.58		
Docetaxel	Yes/No (%Yes)	83 (50.6)	1.45 (0.82 – 2.56)	0.20		
Cabazitaxel	Yes/No (%Yes)	83 (21.7)	1.83 (1.01 – 3.33)	<0.05		
Carboplatin	Yes/No (%Yes)	83 (18.1)	1.52 (0.77 – 2.98)	0.22		
Sipuleucel-T	Yes/No (%Yes)	83 (18.1)	0.69 (0.34 – 1.43)	0.32		
Radium-223	Yes/No (%Yes)	83 (13.3)	0.84 (0.33 – 2.12)	0.71		
Gene Amplification						
AR	Yes or No	83	1.41 (0.80 – 2.48)	0.23		
MYC	Yes or No	83	2.26 (1.24 – 4.12)	<0.01		
BRAF	Yes or No	83	2.38 (1.27 – 4.43)	<0.01		
PIK3CA	Yes or No	83	3.62 (1.83 – 7.18)	<0.01	3.03 (1.5 – 6.11)	<0.01
CDK6	Yes or No	83	2.95 (1.46 – 5.94)	<0.01		
MET2	Yes or No	83	2.81 (1.46 – 5.42)	<0.01		
EGFR	Yes or No	83	4.41 (1.98 – 9.84)	<0.01		
RAF1	Yes or No	83	1.83 (0.78 – 4.31)	0.17		
FGFR1	Yes or No	83	3.48 (1.22 – 9.92)	<0.05		
KIT	Yes or No	83	1.82 (0.72 – 4.60)	0.21		

Assessed by univariate and multivariate Cox proportional hazards models. Only variables found to be statistically significant in the final multivariate model have reported hazard ratio and p-value.

**Figure 6:**
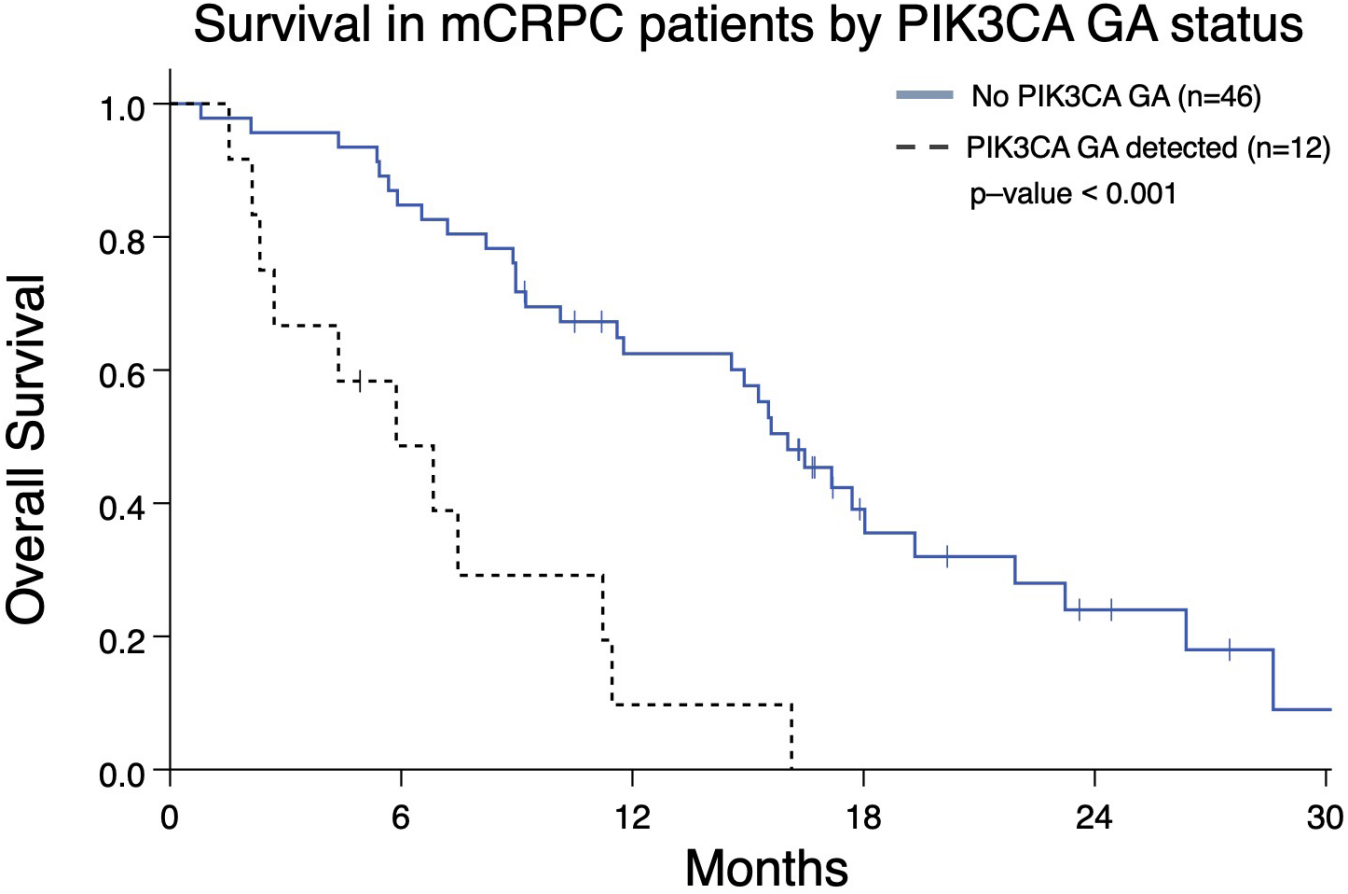
Overall survival from the date of the nearest systemic therapy initialization in Group A patients with mCRPC who have at least one GA (n=58) and either also have or lack a *PIK3CA* GA as well. Patients without *PIK3CA* GA have greater median OS (16.0 months) compared to patients with a *PIK3CA* GA (5.9 months; p < 0.001).

In light of *PIK3CA* GA association with increased liver and lung metastases (**Figure 2D**) and association with worse OS and FFS in multivariate analysis (**Tables 2**, **3**), we determined the impact of a *PIK3CA* amplification on OS when compared to patients with GAs (regardless of number) without *PIK3CA* amplification (**Figure 6**). mCRPC patients that have at least one GA but lack any *PIK3CA* GA was associated with better OS compared to patients whose disease harbors a *PIK3CA* GA (16.0 vs 5.9 months; p-value < 0.001) (**Figure 6**). It is worth noting that a majority of patients with *PIK3CA* GA in this group did have at least one other GA as well (data not shown).

### Reduction in *AR* and/or *MYC* PCN predicts improved OS

**Figure 7:**
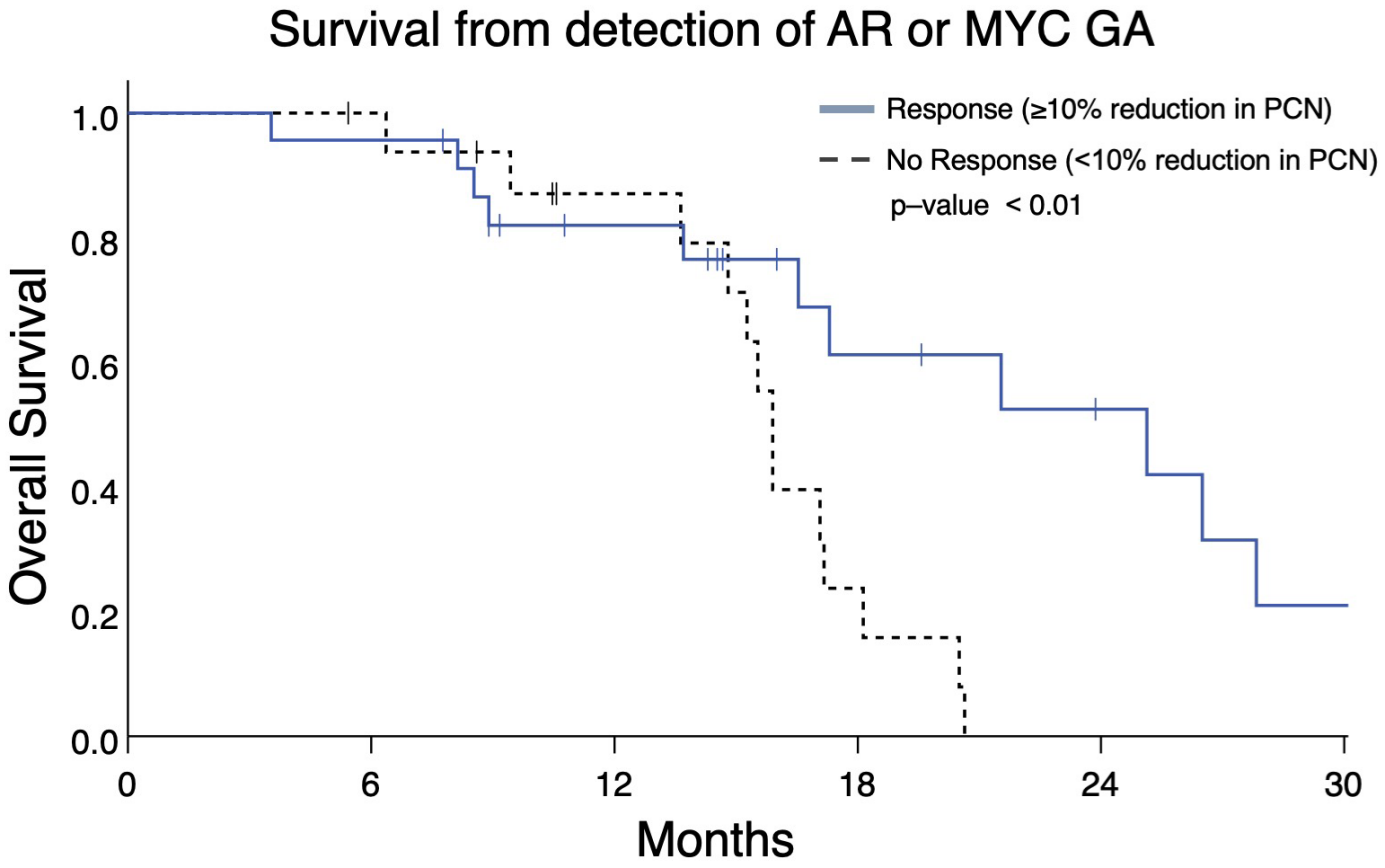
Overall survival in Group B patients with ≥10% reduction in *AR* and/or *MYC* PCN (“Response”) following initial gene amplification had longer survival vs patients without PCN reduction (median OS 25.1 months vs 15.9 months, p = 0.008).

Serial ctDNA profiling permits assessment of PCN changes as they occur during a patient’s treatment course. Therefore, we determined whether changes in PCN in patients with two consecutive ctDNA profiles might be prognostic. Analyzing the two most frequently occurring GAs, we identified patients with mCRPC who had *AR* and/or *MYC* GAs with an additional ctDNA profile completed afterwards to gauge PCN change. These patients were categorized as group B (**Figure 1**). Patients with ≥10% reduction in *AR* and/or *MYC* PCN (termed as “Response”) on the second profile had better OS than those with ≤ 10% *AR* and/or *MYC* PCN decrease (termed as “No Response”) (median OS, 25.1 months vs. 15.9 months, p = 0.008) (**Figure 7**). 

**Figure 8:**
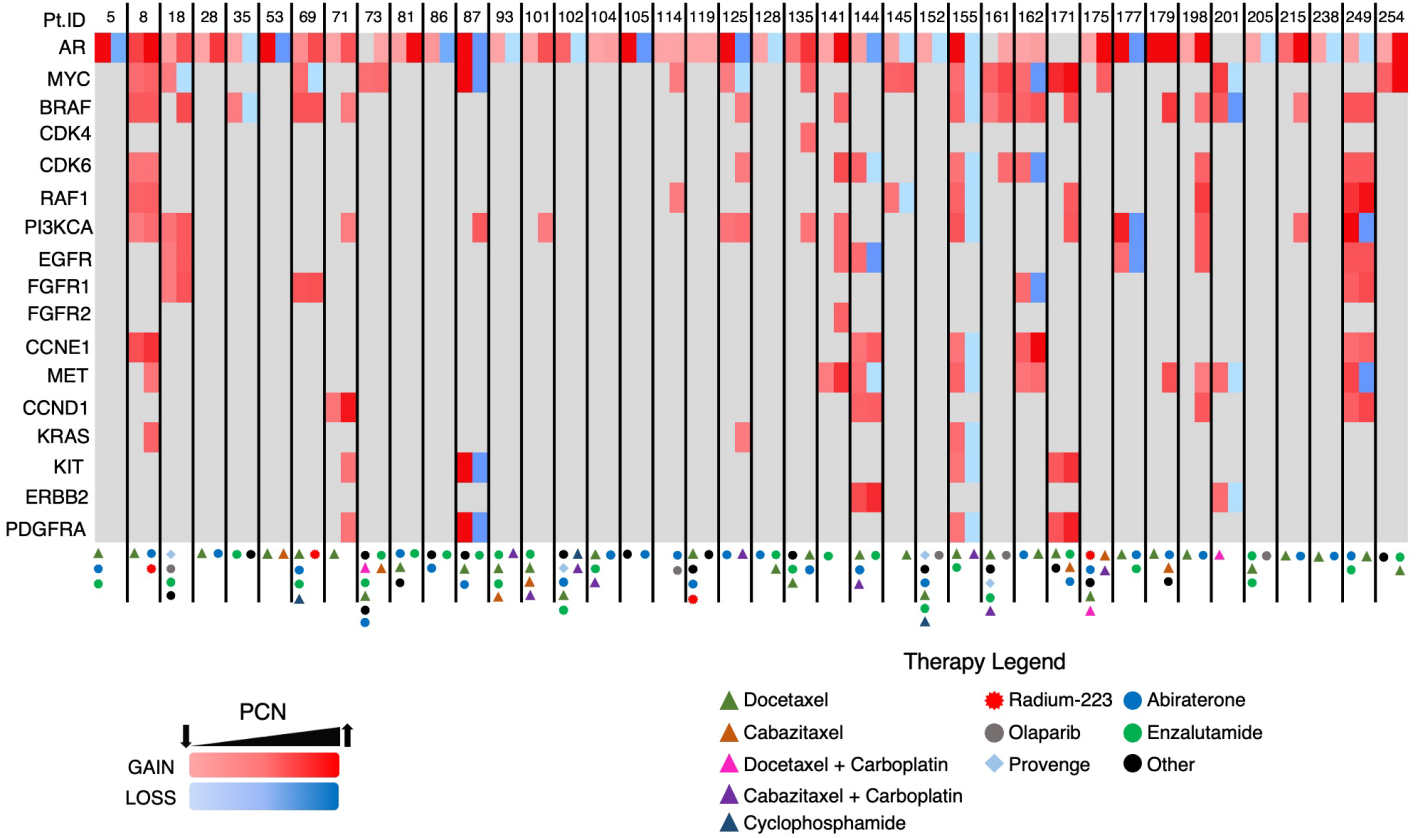
Heatmap of GAs in Group B patients. Each patient had two consecutive ctDNA analyses completed. Two columns are depicted for each patient with the initial *AR*/*MYC* detecting ctDNA analysis in the first column and the subsequent analysis in the second column.

The heatmap in **Figure 8**, which details the GAs identified in group B patients and the treatments those patients underwent prior to each ctDNA analysis, demonstrates PCN reduction following a variety of systemic therapies. Androgen axis-targeting agents such as abiraterone and enzalutamide were associated with detectable *AR*/*MYC* PCN reductions in seven out of 40 patients. Taxane-based chemotherapy (with or without carboplatin) resulted in responses in at least 10 patients. Interestingly, two of the responses were in patients who harbored either a *BRCA2* (PtID 152) or *ATM* mutation (PtID 205) and had *AR* PCN reduction following olaparib administration. In one patient who received Radium-223, *MYC* PCN was reduced while the PCN of other GAs increased (PtID 69), suggesting that perhaps the predominant subclone that was metastatic to the bone in this patient harbored the *MYC* GA and therefore had PCN reduction following Radium-223 treatment. One patient with no identifiable AR mutations was observed to have a reduction in the *MYC* GA-containing subclone following administration of the investigational agent TRC253 (PtID 18). In another patient with an identifiable *BRAF* mutation and amplification (PtID 35), *AR* PCN was reduced after administration of trametinib. Overall, a variety of systemic treatments resulted in PCN reduction of the two predominant GAs in this group and were significantly associated with increased survival.

## Discussion

Circulating tumor DNA analysis is increasingly being used in mPC and its role in decision making is being defined. While many commercially available assays examine the presence of single nucleotide variants, few ctDNA assays report GAs. Our data suggest that clinically useful information may be found in the additional analytes. Furthermore, our findings provide a basis for evaluating ctDNA somatic alterations at diagnosis of metastatic disease and throughout the course of treatment, with an emphasis on identifying GAs and continually assessing changes in their PCN. This paradigm of serial ctDNA analyses might be used not only to guide therapeutic decisions but also inform prognosis and serve as a biomarker of treatment response, complementing known disease markers such as PSA. Of note, prior studies have evaluated early changes of ctDNA across solid tumors and found that patients who achieve ≥50% decrease in their ctDNA ratio early in immunotherapy-based treatment as compared to baseline sampling have a superior PFS and OS advantage compared to patients who do not reach a 50% threshold for decrease (24, 25). While our cohort does contain serial sampling, the timepoints of collection are diverse and are not reflective of an early on-treatment change. Thus, we aimed to demonstrate that any decrease in ctDNA or amplifications in patients with mCRPC, even at a low threshold of 10% reduction, is prognostic of clinical benefit to their therapy.

Previous studies have evaluated the clinical significance of specific mutations and the associations they may have in clonal development and treatment resistance (12–14). Although most patients in prior studies have had detectable co-existing mutations, our study emphasizes evaluation of clinical factors and outcomes in patients stratified based on specific GAs and changes in PCN in prostate cancer. Our approach is supported by the fact that GAs are commonly observed in patients with mPC, particularly when disease is castration-resistant. By identifying specific GAs, ctDNA analysis could prove a valuable management tool for patients seen at various points in their sequential treatment courses.

Inferior outcomes have been associated with alterations in *AR*, such as coding sequence mutations, splice variants, and amplifications (26). With regard to this study, it is important to note findings regarding presence of the number of GAs and which genes may be amplified are nuanced, and need to be evaluated in context (i.e. whether they co-occur with other GAs). For instance, prior work has demonstrated *AR* amplification being associated with reduced FFS and OS (11). However, in our study, “*AR* Plus” patients have worse OS compared to patients exclusively with an *AR* GA (**Figure 5**). This demonstrates *AR* amplification in the presence of other GAs is associated with worse clinical outcomes. In other words, tumor biology in mCRPC may not be as aggressive in a patient with only an *AR* amplification compared to those whose malignancy has multiple GAs that include *AR* (such as *MYC*, *BRAF*, *PIK3CA*, and others).

Increased numbers of GAs are seen in patients with *TP53* mutations (**Figure 2C**). This is consistent with *TP53* mutations being associated with increased aneuploidy frequency in malignancies (27, 28). Previous studies have identified putative associations and roles for *BRAF*, *PIK3CA*, and *FGFR1* in mCRPC (17, 29, 30). The association we found between *BRAF* or *PIK3CA* GAs, and liver or lung metastases, further prioritizes the need to identify potential treatment options for patients with these GAs (**Figure 2D**). This also corresponds to reduced OS seen in patients with *PIK3CA* amplification relative to other patients with GAs (**Table 2** and **Figure 6**). A recent report from our group demonstrated CEA elevation is associated with a number of amplifications including *PIK3CA* (19). However, this was not associated with aggressive variant prostate cancer phenotype (19). Further evaluation regarding the biological input of *PIK3CA* amplification is needed.

Our study identified a high frequency of *BRAF* amplifications compared to the TCGA database. We feel this reflects the advanced disease of the subjects evaluated in this study. As multiple genes are co-amplified with *BRAF* such as *CDK6, EGFR*, and *MET* (also located on chromosome 7), this gives confidence that we are in fact detecting true *BRAF* amplifications. Related to this, we found that *CDK6* amplification was significantly associated with liver/lung metastases; however, it remains to be seen whether this association reveals a significant biological role for *CDK6* in disease pathogenesis or treatment. Finally, we observed an association between *FGFR1* amplification and liver/lung metastases (**Figure 2D**). Pre-clinical studies have demonstrated that *FGFR1* is involved in prostate cancer progression and has a role in the metabolic reprogramming of prostate cancer cells (29, 30).

Serial ctDNA analyses revealed the emergence of GAs in multiple patients following systemic therapy (**Figure 8**, PtID’s 71, 101, 114, 135, and 175). In fact, some patients developed new GAs even after a reduction in *AR* and/or *MYC* PCN (i.e. PtID’s 18, 69, 87, and 125). The latter phenomenon is likely reflective of tumor heterogeneity, with certain clones/subclones sensitive to treatment resulting in PCN reduction, while new subclones then emerge that are resistant to treatment. In the copy number analysis performed on the STAMPEDE patient cohort, sequencing was performed on multiple regions of the same prostate tumor, and intratumoral differences in GA burden, indicating increased heterogeneity, was associated with increased metastatic potential (18).

This study has a number of limitations. It is a single-institution retrospective study intended to be hypothesis-generating in nature and as such does not capture the breadth of geographic differences in patient clinical accessibility, outcomes, and provider bias for ctDNA testing and treatment recommendations. Furthermore, this study had a modest sample size and does not represent the full extent of tumor genomic heterogeneity among patients. Although 155 patients with mPC were evaluated in this cohort, the survival analysis was limited to the 91 subjects in Group A and Group B, (**Figure 1**), who had mCRPC and adequate treatment records for the entire disease course (**Figures 3**, **4**, **7**). Provider bias in the use or sequencing of abiraterone could have affected our finding that it was prognostic for FFS. Although the gene panel used in this study is meant to capture a wide variety of somatic alterations relevant to numerous malignancies, it evaluates a modest number of genes, ranging from 68-74. With regard to mPC, this panel did not evaluate somatic alterations such as *AR* variant 7 splice site changes, *SPOP* mutations, and *PTEN* loss (31, 32). Therefore, these are beyond the scope of this study. Moreover, in the analysis completed in group B, there is variability among patients in the timing between ctDNA analyses. This variability also extends to the timing and variety of systemic treatments administered prior to initial *AR*/*MYC* GA detection and between ctDNA analyses. Future work in controlled trial formats should investigate PCN changes in relevant GAs.

Nonetheless, this study provides significant support for the wider use of ctDNA evaluation to guide prognosis and mPC treatment selection. Additionally, it reinforces the role for serial monitoring of ctDNA to characterize changes in GAs and other somatic alterations. Ultimately, this raises the question whether ctDNA information should be incorporated into decision making regarding early treatment options. For instance, should mPC patients receive more aggressive initial systemic therapy such as the addition of docetaxel to ADT plus androgen axis targeting therapies (i.e. darolutamide or abiraterone as per the ARASENS or PEACE-1 studies) if they harbor GAs that are linked to increased likelihood of visceral metastases (33, 34)? Ongoing prospective clinical studies are evaluating the impact of pretreatment ctDNA evaluation on therapeutic decisions and clinical outcomes in metastatic disease. Additionally, it remains unknown whether specific amplifications are associated with increased expression of their respective gene products and enhanced downstream signaling of relevant oncogenic pathways. If such an association exists, protein products of select GAs would make attractive therapeutic targets.

Findings from our retrospective study demonstrate that GAs, as detected by ctDNA analysis, may play a significant role in informing potential risk for visceral metastases and overall prognosis in mCRPC. Furthermore, evaluation of serial PCN during mCRPC treatment provides an additional tool to determine putative responses to a variety of therapeutic interventions and, as such, can supplement clinical decision-making. Future studies are needed to determine the prognostic significance of specific GAs and the reliability of using PCN as a clinical tool to assess treatment responses.

## Data availability statement

The datasets presented in this article are not readily available because of patient anonymity. Requests to access the datasets should be directed to the corresponding author.

## Ethics statement

The studies involving humans were approved by Medical University of South Carolina IRB. The studies were conducted in accordance with the local legislation and institutional requirements. Written informed consent for participation was not required from the participants or the participants’ legal guardians/next of kin in accordance with the national legislation and institutional requirements.

## Author contributions

TD contributed to conceptualization, data analysis, study design, and was the primary manuscript writer/editor. ML contributed to conceptualization, data analysis, study design, and manuscript writing/editing. HL performed formal statistical analysis and manuscript review/editing. JK, LD, and AG contributed to data analysis, and manuscript writing/editing. TG and PH contributed to manuscript review/editing. All authors contributed to the article and approved the submitted version.
